# Impact of tillage and crop establishment methods on rice yields in a rice-ratoon rice cropping system in Southwest China

**DOI:** 10.1038/s41598-021-98057-x

**Published:** 2021-09-16

**Authors:** Peng Jiang, Fuxian Xu, Lin Zhang, Mao Liu, Hong Xiong, Xiaoyi Guo, Yongchuan Zhu, Xingbing Zhou

**Affiliations:** 1 Rice and Sorghum Research Institute, Sichuan Academy of Agricultural Sciences/Key Laboratory of Southwest Rice Biology and Genetic Breeding, Ministry of Agriculture, Deyang, 618000 China; 2 Crop Ecophysiology and Cultivation Key Laboratory of Sichuan Province, Wenjiang, 611130 China

**Keywords:** Plant regeneration, Plant physiology

## Abstract

Simplified cultivation methods for rice production offer considerable social, economic, and environmental benefits. However, limited information is available on yield components of rice grown using simplified cultivation methods in a rice-ratoon rice cropping system. A field experiment using two hybrid and two inbred rice cultivars was conducted to compare four cultivation methods (conventional tillage and transplanting, CTTP; conventional tillage and direct seeding, CTDS; no-tillage and transplanting, NTTP; no-tillage and direct seeding, NTDS) in a rice-ratoon rice system from 2017 to 2020. Main season yields for CTDS and NTDS were higher than for CTTP by 6.1% and 2.8%, respectively; whereas ratoon season yields for CTDS and NTDS were equal to or higher than for CTTP. Annual grain yields for CTDS and NTDS were higher than for CTTP by 4.4% and 3.2%, respectively. The higher CTDS and NTDS yields were associated with higher panicle numbers per m^2^ and biomass production. Rice hybrids had higher yields than inbred cultivars by 15.8–19.3% for main season and by 15.6–19.4% for ratoon season, which was attributed to long growth duration, high grain weight and biomass production. Our results suggest that CTTP can be replaced by CTDS and NTDS to maintain high grain yields and save labor costs. Developing cultivars with high grain weight could be a feasible approach to achieve high rice yields in the rice-ratoon rice cropping system in southwest China.

## Introduction

Rice is the main staple food crop that supports a large segment of the global population^1^. It has been estimated that global rice production must increase by 116 million tons by 2035 to meet the growing demand for food that will result from predicted population growth^2^. In addition, China will need to produce approximately 20% more rice by 2030 to meet its projected domestic needs^3^. Further increases in rice production in China are mainly dependent on more frequent harvests on the existing cropland^4,5^. In recent years, the area of arable cropland used for rice production has been decreasing because of increasing use of land for urbanization^6^. Yield gap analysis has also shown that rice yields have been approaching their potential biophysical ceiling^7,8^, and the average annual growth rate was − 0.3% from 1998 to 2006 in China^9^. In general, multiple rice cropping systems include double-season rice, triple-season rice, and ratoon rice; the double-season rice system is the dominant multiple rice cropping system used in China and elsewhere in Asia^10^. However, the planting area of the double rice cropping systems in China has continued to decline because of labor shortages, a low level of mechanization, and low production efficiency^11^. To ensure rice self-sufficiency and food security, strategies to increase the productivity of multiple rice cropping systems are desperately needed.

**Figure 1:**
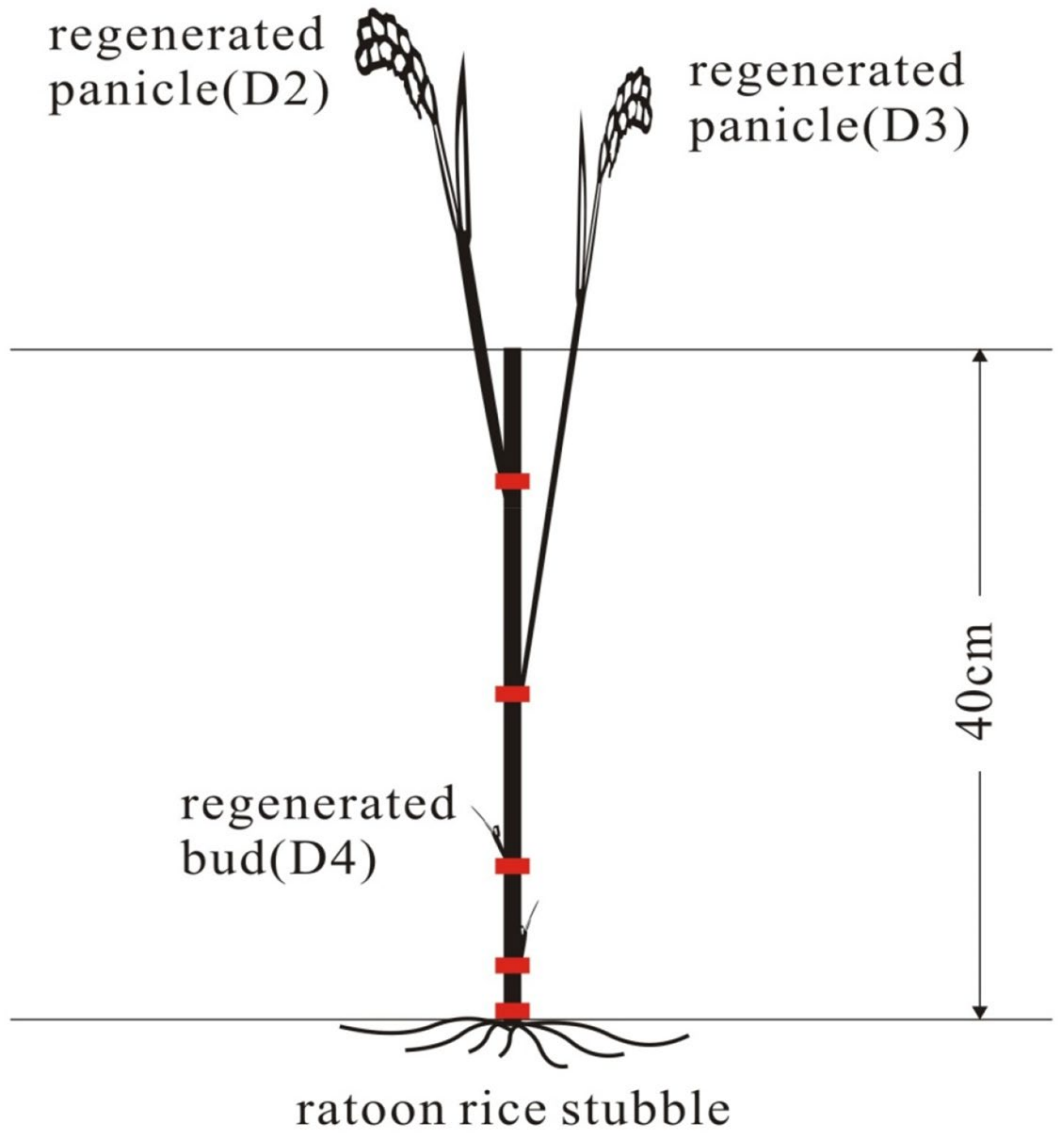
The brief diagram of the nodes, regenerated panicles and regenerated buds in ratoon rice system. D2, D3 and D4 represent the second node from top, third node from the top, and other nodes below the third node from main crop tillers.

Ratoon rice (Fig. 1), which is the practice of obtaining a second harvest from tillers originating from the stubble remaining after harvesting the main rice crop, has emerged as an alternative option to replace double-season rice^12^. This is called a "one planting and two harvests" cropping system. Ratoon rice crops are characterized by short growth periods, high daily yields, high rice quality, low labor requirements, low water usage, reduced seed usage, reduced production costs, and increased yield and profits^13^. With progress in rice breeding and improvement crop management practices, and shortage of labor, use of rice ratooning has rapidly expanded in China^14,15^, the total planted area of ratoon rice cultivation was more than 1.24 million hectares in 2020^16^. The practice of ratoon rice has also rapidly increasing in USA and Africa and elsewhere in world^17–19^. The grain yield of ratoon rice is more than 6.0 t ha^−1^ if the crop is properly managed^20^.

At present, conventional tillage is the most widely used method for land preparation of paddy fields for the main season rice crop. In ratoon rice systems, the crop establishment method used for the main season crop is mainly seedling transplantation, which is still the dominant method used in China. Transplanting seedlings requires a large amount of manpower and the task is very labor-intensive and involves working in a stooping posture and moving in a muddy field^21^. Paradoxically, labor availability is limited in China because an increasing number of young farmers have left for jobs in the cities, leaving the older farmers behind. Recently, simplified cultivation technologies for rice production have become increasingly attractive because the potential benefits include savings on labor, reduced water use, lower greenhouse gas emissions, and reduced environmental risks as well as improved rice yield if the crop is properly managed^22^.

Simplified cultivation technologies for rice production commonly rely on simplifying the methods used for crop establishment, or land preparation, or both. At present, several simplified cultivation technologies, such as conventional tillage and direct seeding (CTDS), no-tillage and direct seeding (NTDS), conventional tillage and seedling broadcasting (CTSB), no-tillage and seedling broadcasting (NTSB), and no-tillage and transplanting (NTTP), have been developed in China for rice production. The adoption of simplified cultivation technologies for producing the main rice crop in place of conventional transplanting is continuously increasing in Asia, because this can potentially reduce production costs through savings on fuel and labor while maintaining grain yields and increasing system productivity and resource use efficiency compared with conventional transplanting^23,24^. However, the effects of simplified cultivation technologies on rice yields in the rice-ratoon rice cropping system is still unknown.

Rice yield is determined by the number of panicles per unit land area, the spikelet number per panicle, the spikelet filling percentage, and grain weight. Lin et al. showed that the number of spikelets per panicle contributed the most to grain yield in the main rice crop, and the number of panicles per unit area of land contributed the most to grain yield in ratoon rice through the sink size (spikelet number per unit land area)^25^. The grain yield of ratoon rice is significantly and positively correlated with the number of panicles per unit land area^26^. The largest planting area of ratoon rice in China is in southwest China, and accounts for 40% of the current total land area devoted to ratoon rice. However, rice farmers transplant rice seedlings at extremely wide spacing in southwest China due to the shortage of rural labor and increased labor costs^3^. Grain yields of the main rice crop and ratoon rice in the farmers' paddy fields planted at low density were only 93% and 70%, respectively, of the yield from 666 hectares of demonstration fields planted at high density; and the average total yield in the farmers' paddy fields was approximately 1.5 t ha^−1^ lower than that in the demonstration fields^13^. These results indicate that the number of panicles per unit land area plays a vital role in yield formation in ratoon rice, which depends on the ability of the meristematic buds to regenerate, or the number of mother stems per unit land area, or both. The ability of the buds to regenerate is affected by both genetics and management practice factors. Xu et al. has suggested that the selection of genotypes with strong bud regeneration ability in breeding programs could be an effective way to achieve an increase in panicle number per unit land area in the ratoon season crop^13^. Huang et al. reported that grain yield in the direct-seeded (DS) main rice crop was higher than in the main crop using conventional transplanting (CT), and that this was mainly due to the higher panicle number per m^2^ of the main rice crop^27^. These results imply that the panicle number per m^2^ in the ratoon season can be increased by increasing the number of rice mother stems through the use of DS. However, limited information is available on grain yield and yield components in rice grown using simplified cultivation technologies in the rice-ratoon rice cropping system. A four-year field experiment was conducted with the following aims: (1) to examine the effects of simplified cultivation technologies on grain yield in the rice–ratoon rice cropping system; (2) to the determine yield formation characteristics of rice grown using simplified cultivation technologies in the rice–ratoon rice cropping system.

## Results

### Crop growth and development

The length of the ratoon growth season was 45–48% that of main season (Table 1). The lengths of the main and ratoon growing seasons were similar for the four years of the experiment. Hybrid rice cultivars with longer main growing seasons also had longer ratoon growing seasons. The total main season growth durations for the rice hybrids were 9–10 days longer than for the inbred rice cultivars. The longer total main season growth duration of the rice hybrids was mainly attributed to the longer growing time from sowing to heading. Similarly, the total growth duration for the ratoon season was longer for the rice hybrids than for the inbred rice cultivars by 4–7 days.

**Table1:** Growth duration of four rice cultivars in main rice crop -ratoon rice system in 2017–2020.

Cultivar	Year	SW-HD^a^ (d)	HD-MH (d)	Main crop duration (d)	MH-HDR (d)	HDR-RH (d)	Ratoon crop duration (d)
HHZ & JNSM^b^	2017	112	30	142	34	30	64
	2018	110	30	140	32	32	64
	2019	111	30	141	34	30	64
	2020	111	30	141	34	31	65
NY103 & RY1015	2017	119	32	151	38	32	70
	2018	117	32	149	38	33	71
	2019	118	33	151	37	31	68
	2020	117	33	150	39	33	72

^a^SW-HD, from sowing to heading; HD-MH, from heading to harvest of the main rice crop; MH-HDR, from harvest of the main rice crop to heading of the ratoon rice; HDR-RH, from heading of the ratoon rice to harvest of the ratoon rice.

^b^HHZ, JNSM, NY103 and RY015 are Huanghuazhan, Jinnongsimiao, Nei6you103 and Rongyou1015, respectively.

### Climatic condition

The maximum and minimum temperatures were slightly higher for the rice hybrids from sowing (SW) to heading (HD) and from HD to harvest for the main rice crop (MH) than for the rice inbreds during the main growing season (Table 2). However, the rice hybrids experienced lower maximum and minimum temperatures from MH to heading of the ratoon rice (HDR) and from HDR to harvest of the ratoon rice (RH) than did the rice inbred lines in the ratoon growing season. The solar radiation was 1.7% lower for the rice hybrids from SW to HD than for the rice inbreds during the main growing season, while there was 3.9% higher solar radiation for the rice hybrids from HD to MH than for the rice inbreds. Rice hybrids had lower solar radiation than did rice inbreds by 10.6% from MH to HDR and by 13.6% from HDR to RH during the ratoon rice growing season.

**Table 2:** Maximum temperature, minimum temperature and solar radiation during the rice-growing season from sowing to maturity of ratoon rice in 2017–2020.

Cultivar	Year	SW-HD^a^	HD-MH	MH-HDR	HDR-RH
**Maximum temperature (°C)**					
HHZ & JNSM ^b^	2017	25.9	35.2	33.0	26.7
	2018	27.5	34.1	34.6	24.3
	2019	26.0	31.1	33.3	27.1
	2020	26.9	30.7	33.8	24.6
	Mean	26.6	32.8	33.7	25.7
NY103 & RY1015	2017	26.3	35.6	31.6	22.9
	2018	27.6	35.1	31.2	21.5
	2019	26.1	32.1	31.8	24.9
	2020	27.0	32.4	30.8	20.8
	Mean	26.7	33.8	31.3	22.5
**Minimum temperature (°C)**					
HHZ & JNSM	2017	18.0	26.2	24.6	21.0
	2018	19.0	25.6	25.3	20.1
	2019	18.4	23.9	24.4	20.2
	2020	18.5	24.1	24.7	19.8
	Mean	18.5	24.9	24.7	20.2
NY103 & RY1015	2017	18.4	26.2	23.8	18.4
	2018	19.4	25.5	23.8	17.3
	2019	18.6	24.7	23.5	18.3
	2020	18.8	25.0	22.8	17.1
	Mean	18.8	25.3	23.5	17.8
Solar radiation (MJ m^−2^ d^−1^)					
HHZ & JNSM	2017	17.1	19.4	17.3	12.2
	2018	18.1	18.8	18.5	10.6
	2019	16.2	17.2	17.8	13.4
	2020	17.4	16.2	18.1	11.1
	Mean	17.2	17.9	17.9	11.8
NY103 & RY1015	2017	17.0	19.7	15.9	10.0
	2018	16.2	18.8	15.6	9.5
	2019	15.7	17.4	16.4	12.2
	2020	16.9	17.2	16.1	8.9
	Mean	16.5	18.3	16.0	10.2

^a^SW-HD, from sowing to heading; HD-MH, from heading to harvest of the main rice crop; MH-HDR, from harvest of the main rice crop to heading of the ratoon rice; HDR-RH, from heading of the ratoon rice to harvest of the ratoon rice.

^b^HHZ, JNSM, NY103 and RY015 are Huanghuazhan, Jinnongsimiao, Nei6you103 and Rongyou1015, respectively.

### Grain yield and its attributes

Main season yield significantly differed among cultivation method treatments (T) and rice cultivars (C), whereas the interactive effects of T × C were not significant for main season yield (except 2019) (Table 3). The effects of T, C and their interaction were significantly on ratoon season yield in 2018 but were not significant in 2017. Ratoon season yield was significantly affected by C in 2019 and 2020, but not by T and the interaction of T × C. Annual yield significantly differed among T and C. The interactive effect of T × C was significant for annual yield in 2018 and 2019 but was not significant in 2017 and 2020.

**Table 3:** Main season yield, ratoon season yield and annual yield of four rice cultivars grown under four cultivation methods in 2017–2020.

Treatment	cultivar	2017			2018			2019			2020		
		Main yield	Ratoon yield	Annual yield	Main Yield	Ratoon yield	Annual yield	Main yield	Ratoon yield	Annual yield	Main yield	Ratoon yield	Annual yield
CTDS	HHZ	10.17 c	3.15 bc	13.32 c	8.89 c	2.28 b	11.17 c	8.24 b	2.87 c	11.11 b	7.91 b	3.27 c	11.18 b
	JNSM	9.80 c	2.91 c	12.71 c	8.34 c	2.57 b	10.90 c	7.87 b	3.34 b	11.21 b	7.86 b	3.09 c	10.94 b
	NY103	12.12 a	3.63 a	15.75 a	9.54 b	2.84 b	12.39 b	9.46 a	3.63 a	13.09 a	8.98 a	4.07 a	13.05 a
	RY1015	11.28 b	3.49 ab	14.77 b	10.14 a	3.53 a	13.67 a	9.42 a	3.24 b	12.66 a	9.35 a	3.60 b	12.95 a
	Mean	10.84 A	3.29 AB	14.13 AB	9.23 A	2.80 AB	12.03 A	8.75 A	3.27 A	12.02 A	8.52 A	3.51 A	12.03 A
NTDS	HHZ	10.26 b	3.60 a	13.86 ab	8.64 ab	2.14 c	10.79 b	7.53 c	2.84 c	10.36 b	7.37 b	3.33 c	10.7 b
	JNSM	9.57 b	3.49 a	13.06 b	8.13 b	2.78 b	10.91 b	7.70 bc	3.42 a	11.12 ab	7.90 b	3.23 c	11.13 b
	NY103	11.68 a	3.44 a	15.12 a	9.36 ab	4.43 a	13.79 a	8.64 ab	3.36 ab	12.00 a	8.84 a	4.26 a	13.10 a
	RY1015	11.41 a	3.49 a	14.89 a	9.71 a	3.03 b	12.75 a	8.89 a	3.27 b	12.16 a	9.20 a	3.69 b	12.88 a
	Mean	10.73 AB	3.50 A	14.23 A	8.96 A	3.10 A	12.06 A	8.19 B	3.22 A	11.41 B	8.32 A	3.63 A	11.95 A
NTTP	HHZ	9.61 b	3.19 a	12.79 c	7.9 b	2.14 b	10.04 b	7.19 b	3.05 c	10.24 b	7.68 b	3.21 b	10.89 b
	JNSM	9.45 b	3.07 a	12.53 c	7.34 b	2.43 b	9.77 b	7.08 b	3.30 bc	10.39 b	6.89 c	3.36 b	10.25 c
	NY103	11.63 a	3.41 a	15.04 a	8.62 a	3.3 a	11.91 a	9.11 a	3.82 a	12.93 a	8.25 ab	4.05 a	12.31 a
	RY1015	11.06 a	3.09 a	14.15 b	9.03 a	3.1 a	12.12 a	8.90 a	3.41 b	12.31 a	8.70 a	3.27 b	11.97 a
	Mean	10.44 B	3.19 B	13.63 B	8.22 B	2.74 AB	10.96 B	8.08 B	3.39 A	11.47 AB	7.88 B	3.47 A	11.35 B
CTTP	HHZ	9.85 c	3.48 a	13.32 c	8.03 b	2.04 c	10.07 b	7.62 b	2.99 c	10.62 b	8.06 bc	3.30 b	11.36 b
	JNSM	9.45 c	3.40 a	12.86 c	7.65 b	2.16 c	9.80 b	6.30 c	3.24 b	9.54 c	7.77 c	3.30 b	11.07 b
	NY103	11.37 a	3.28 a	14.65 a	8.64 a	3.73 a	12.38 a	9.29 a	3.63 a	12.92 a	8.68 ab	4.21 a	12.89 a
	RY1015	10.74 b	3.27 a	14.01 b	9.15 a	2.77 b	11.92 a	8.99 a	3.35 b	12.33 a	9.25 a	3.42 b	12.67 a
	Mean	10.35 B	3.36 AB	13.71 B	8.37 B	2.67 B	11.04 B	8.05 B	3.30 A	11.35 B	8.44 A	3.56 A	12.00 A
Analysis of variance													
Treatment		**	ns	**	**	*	**	**	ns	**	**	ns	**
Cultivar		**	ns	**	**	**	**	**	**	**	**	**	**
T × C		ns	ns	ns	ns	**	*	*	ns	*	ns	ns	ns

^a^CTDS, NTDS, NTTP and CTTP represent conventional tillage and direct seeding, no-tillage and direct seeding, no-tillage and transplanting and conventional tillage and transplanting respectively.

^b^HHZ, JNSM, NY103 and RY015 are Huanghuazhan, Jinnongsimiao, Nei6you103 and Rongyou1015, respectively. Within a column means followed by the same letter are not significantly different according to LSD at P = 0.05. Lower-case and upper-case letters indicate comparison among four Cultivars and between four cultivation treatments, respectively. *Significant at *P* < 0.05, **Significant at *P* < 0.01, ns, not significant at *P* < 0.05.

Mean main season yield across four rice cultivars and four years under CTDS and NTDS treatments reached to 9.34 and 9.05 t ha^−1^, respectively, which were approximately 6.1% and 2.8% higher than the yields under CTTP treatment, respectively (Table 3). However, grain yield of ratoon rice for the CTDS and NTDS treatments was equal to or higher than for the CTTP treatment. Consequently, the annual grain yield for rice grown using CTDS and NTDS treatments was 4.4% and 3.2% higher, respectively, than for rice grown using CTTP treatment. Main season yield, ratoon season yield, and annual grain yield for the NTTP treatment were equal to or higher than for the CTTP treatment.

A large difference was observed in main season yield and ratoon season yield between the rice hybrids and the inbred cultivars (Table 3), though the magnitude of the difference in main season yield and ratoon season yield between the rice hybrids and the inbred cultivars varied by cultivation method. Average across four years, the rice hybrids had higher main season yields than did the rice inbreds by 16.2% under CTDS, by 15.8% under NTDS, by 19.3% under NTTP and by 17.6% under CTTP. The main season yield gap between the hybrid and inbred rice were larger in transplanting treatment. The ratoon season yields for rice hybrids were higher than for rice inbreds by 19.4% under CTDS, by 16.7% under NTDS, by 15.6% under NTTP and by 15.7% under CTTP. The ratoon season yield gap between the hybrid and inbred rice were larger in direct seeding treatment. The difference in annual yield among four rice cultivars were significant in all four experiments. On average, the hybrids had 17.1% higher annual grain yield than did the inbred cultivars.

The main season yields were 21.8%, 28.1%, and 27.7% higher in 2017 than in 2018, 2019, and 2020, respectively. The ratoon season yield was similar to, or slightly higher in 2017, than it was in 2018, 2019, and 2020. As a result, the annual grain yields were 20.8%, 20.5%, and 17.7% higher than in 2018, 2019, and 2020, respectively.

The yield components of main rice crop were significantly affected by year (Y), cultivation method treatment (T) and cultivar (C), except the effect of T for grain weight (Table 4). The interactive effects of Y × T, T × C and Y × T × C were significant for panicles per m^2^ and spikelets per panicle in the main season rice crop but were not significant for grain filling and grain weight in the main rice crop. There was a significant Y × C interactive effect on panicles per m^2^, spikelets per panicle, spikelets per m^2^, grain filling, and grain weight in the main rice crop.

**Table 4:** Yield components of four rice cultivars grown under four cultivation methods in main crop season in 2017–2020.

Variable	Panicles per m^2^	Spikelets per panicle	× 10^3^ Spikelets per m^2^	Grain filling (%)	Grain weight (mg)
Treatment (T)^a^					
CTDS	354.2 a	121.4 d	42.3 a	90.3 b	24.9 a
NTDS	327.9 b	130.2 c	41.7 a	91.3 a	24.9 a
NTTP	230.7 d	159.9 a	36.2 c	91.3 a	24.7 a
CTTP	242.8 c	154.6 b	37.1 c	91.5 a	24.8 a
Cultivar (C)^b^					
HHZ	330.3 a	133.4 b	42.1 b	92.0 b	21.9 c
JNSM	263.0 d	176.7 a	45.2 a	88.6 d	19.6 d
N6Y103	287.1 b	121.9 c	34.1 d	93.2 a	29.6 a
RY1015	275.2 c	134.1 b	36.0 c	90.6 c	28.1 b
Year (Y)					
2017	331.4 a	138.1 b	44.1 a	92.0 a	25.2 a
2018	279.1 b	140.3 b	37.5 c	92.6 a	24.6 c
2019	273.0 b	138.1 b	36.2 d	89.6 b	24.9 b
2020	272.1 b	149.6 a	39.6 b	90.2 b	24.6 c
Analysis of variance					
Year (Y)	**	**	**	**	**
Treatment (T)	**	**	**	*	ns
Cultivar (C)	**	**	**	**	**
Y × T	**	**	ns	ns	ns
Y × C	**	**	**	**	**
T × C	**	**	*	ns	ns
Y × T × C	**	**	**	ns	ns

^a^CTDS, NTDS, NTTP and CTTP represent conventional tillage and direct seeding, no-tillage and direct seeding, no-tillage and transplanting and conventional tillage and transplanting respectively.

^b^HHZ, JNSM, NY103 and RY015 are Huanghuazhan, Jinnongsimiao, Nei6you103 and Rongyou1015, respectively. Means within each variable sharing the same letter are not significantly different according to LSD at P = 0.05. *Significant at *P* < 0.05, ** Significant at *P* < 0.01, ns, not significant at *P* < 0.05.

The panicles per m^2^ in the main rice crop for the CTDS and NTDS treatments were 45.8% and 35.0% higher than for the CTTP treatment, respectively, and there were 21.5% and 15.8% fewer spikelets per panicle in the main rice crop for the CTDS and NTDS treatments than for the CTTP treatment, respectively (Table 4). Consequently, the spikelets per m^2^ in the main rice crop for the CTDS and NTDS treatments were 14.0% and 12.4% higher than for the CTTP treatment, respectively. The number of panicles per m^2^ in the main rice crop for the NTTP treatment was 5.0% lower than for the CTTP treatment; however, the number of spikelets per panicle for main rice crop was 3.4% higher for the NTTP treatment compared to CTTP. There was not significantly difference in spikelets per m^2^ between NTTP and CTTP treatment. The differences in grain filling and grain weight in the main rice crop among the four cultivation methods were relatively small.

The inbred cultivar ‘Huanghuazhan’ had the highest number of panicles per m^2^ and ‘Jinnongsimiao’ had the most spikelets per panicle in the main growing season (Table 4). On average, the rice hybrids had 5.2% fewer panicles per m^2^, 17.4% fewer spikelets per panicle and 19.7% fewer spikelets per m^2^ than did the rice inbreds in the main growing season, while grain filling and grain weight were 1.7% and 39.0% higher, respectively, in the rice hybrids compared to the inbred cultivars.

The panicles per m^2^, spikelets per m^2^, and grain weight in the main rice crop were higher in 2017 than in 2018, 2019, and 2020 (Table 4). The number of spikelets per panicle in the main rice crop was slightly lower in 2017 than in 2018, 2019, and 2020. The grain filling in the main rice crop was 0.6% lower in 2017 than in 2018 but 2.0–2.7% higher than 2019 and 2020.

The yield components of ratoon rice were significantly affected by year (Y), cultivation method treatment (T) and cultivar (C), except effect of T for grain weight (Table 5). The interactive effects of Y × T and Y × C were significant for yield components in the ratoon rice crop except for the interactive effect of Y × T for spikelets per panicle and grain weight. The interactive effect of T × C was not significant for number of panicles per m^2^, spikelets per panicle, spikelets per m^2^ or grain filling in ratoon rice but was significant for grain weight in ratoon rice. The interactive effect of Y × T × C was not significant for the number spikelets per panicle, number spikelets per m^2^, grain filling, or grain weight in ratoon rice but was significant for the number of panicles per m^2^ in ratoon rice.

**Table 5:** Yield components of four rice cultivars grown under four cultivation methods in the ratoon rice season in 2017–2020.

Variable	Panicles per m^2^	Spikelets per panicle	× 10^3^ Spikelets per m^2^	Grain filling (%)	Grain weight (mg)
Treatment (T)^a^					
CTDS	352.2 a	55.8 b	18.5 a	72.9 b	21.1 a
NTDS	327.3 b	59.3 a	18.8 a	73.4 b	21.3 a
NTTP	279.4 c	61.4 a	16.8 b	76.9 a	21.1 a
CTTP	288.7 c	61.0 a	17.1 b	76.9 a	21.1 a
Cultivar (C)^b^					
HHZ	338.7 a	58.0 b	19.2 a	76.3 b	17.5 c
JNSM	291.5 b	67.3 a	18.7 a	80.8 a	16.5 d
N6Y103	342.5 a	51.4 c	17.1 b	71.7 c	25.4 a
RY1015	274.9 b	60.7 b	16.1 b	71.2 c	25.1 b
Year (Y)					
2017	385.4 a	64.1 b	24.4 a	72.0 b	21.3 b
2018	322.0 b	54.2 c	17.2 b	71.4 b	20.1 c
2019	337.2 b	45.6 d	15.1 c	78.6 a	22.0 a
2020	203.0 c	73.6 a	14.5 c	78.1 a	21.1 b
Analysis of variance					
Year (Y)	**	**	**	**	**
Treatment (T)	**	**	**	**	ns
Cultivar (C)	**	**	**	**	**
Y × T	*	ns	*	**	ns
Y × C	**	**	**	**	**
T × C	ns	ns	ns	ns	*
Y × T × C	*	ns	ns	ns	ns

^a^CTDS, NTDS, NTTP and CTTP represent conventional tillage and direct seeding, no-tillage and direct seeding, no-tillage and transplanting and conventional tillage and transplanting respectively.

^b^HHZ, JNSM, NY103 and RY015 are Huanghuazhan, Jinnongsimiao, Nei6you103 and Rongyou1015, respectively. Means within each variable sharing the same letter are not significantly different according to LSD at P = 0.05. *Significant at *P* < 0.05, **Significant at *P* < 0.01, ns, not significant at *P* < 0.05.

The number of panicles per m^2^ in ratoon rice for the CTDS and NTDS treatments were 21.9% and 13.4% higher, respectively, than in the CTTP treatment, while there were 8.5% and 2.7% fewer spikelets per panicle, respectively, in ratoon rice in the CTDS and NTDS treatments compared to CTTP (Table 5). Consequently, the spikelets per m^2^ in ratoon rice for the CTDS and NTDS treatments were 8.2% and 9.9% higher, respectively, than in the CTTP treatment. Grain filling in ratoon rice in the CTDS and NTDS treatments was lower than in the CTTP treatment by 5.2% and 4.6%, respectively. The differences in yield components of ratoon rice between the CTTP and NTTP treatments were relatively small, and there were no significant differences in grain weight among the four cultivation methods.

The hybrid ‘Nei6you107’ had the highest number of panicles per m^2^ and the cultivar ‘Jinnongsimiao’ had the most spikelets per panicle in the ratoon season (Table 5). On average, rice hybrids had fewer panicles per m^2^, fewer spikelets per panicle, fewer spikelets per m^2^ and less grain filling than the inbred cultivars by 2.0%, 10.5%, 12.4% and 9.0% in the ratoon season, respectively, while grain weight in the rice hybrids was 48.5% higher than in the inbred lines in the ratoon season.

The number of panicles per m^2^ and spikelets per m^2^ were the highest in 2017, and the number of spikelets per panicle was the highest in the 2020 in ratoon season (Table 5). Grain filling in ratoon rice in 2017 and 2018 was significantly lower than it was in 2019 and 2020. The differences in grain weight in ratoon rice among the four years were relatively small or inconsistent.

### Biomass production and harvest index

Total dry matter and harvest index in the main season rice crop were significantly affected by year (Y), cultivation method treatment (T) and cultivar (C) (Table 6). The interactive effects of Y × T, Y × C, T × C, and Y × T × C were significant for total dry matter and harvest index in the main season rice crop. The total dry matter of the main rice crop in the CTDS and NTDS treatments was higher than in the CTTP treatment by 15.9% and 14.7%, respectively, whereas the harvest indexes of the main rice crop in the CTDS and NTDS treatments were lower than in the CTTP treatment by 2.4% and 1.8%, respectively. The NTTP treatment produced 4.5% less total dry matter in the main rice crop than the CTTP treatment; however, the harvest index of the main rice crop in the NTTP treatment was 1.7% higher than in the CTTP treatment. The rice hybrids had 12.6% higher total dry matter compared to the inbred cultivars in the main season. The difference in the harvest index for the main rice crop between the hybrids and the inbred cultivars was relatively small. The total dry matter was highest in 2017 and the harvest index was highest in 2020 in the main growing season.

**Table 6:** Biomass and harvest index of four rice cultivars grown four cultivation methods in main crop-ratoon rice system in 2017–2020.

Variable	Main season		Ratoon season	
	Total dry matter (g m^−2^)	Harvest index (%)	Total dry matter (g m^−2^)	Harvest index (%)
Treatment (T)^a^				
CTDS	1767.0 a	52.9 c	654.6 a	42.8 c
NTDS	1748.6 a	53.2 c	660.9 a	43.2 bc
NTTP	1455.7 c	55.1 a	586.4 b	44.4 ab
CTTP	1524.5 b	54.2 b	603.1 b	44.7 a
Cultivar (c)^b^				
HHZ	1581.2 c	54.0 a	600.4 b	42.4 c
JNSM	1474.1 d	53.4 b	544.9 c	45.7 a
N6Y103	1738.7 a	54.1 a	722.1 a	43.2 bc
RY1015	1701.8 b	53.9 ab	637.6 b	43.8 b
Year (Y)				
2017	1884.0 a	53.4 b	763.9 a	47.4 a
2018	1581.1 b	53.4 b	576.4 c	42.6 c
2019	1488.3 d	53.1 b	629.2 b	41.0 d
2020	1542.4 c	55.6 a	535.6 d	44.1 b
Analysis of variance				
Year (Y)	**	**	**	**
Treatment (T)	**	**	**	**
Cultivar (C)	**	*	**	**
Y × T	**	**	**	**
Y × C	**	**	**	**
T × C	**	*	ns	ns
Y × T × C	**	**	**	**

^a^CTDS, NTDS, NTTP and CTTP represent conventional tillage and direct seeding, no-tillage and direct seeding, no-tillage and transplanting and conventional tillage and transplanting respectively. ^b^HHZ, JNSM, NY103 and RY015 are Huanghuazhan, Jinnongsimiao, Nei6you103 and Rongyou1015, respectively. Means within each variable sharing the same letter are not significantly different according to LSD at P = 0.05. *Significant at *P* < 0.05, **Significant at *P* < 0.01, ns, not significant at *P* < 0.05.

Total dry matter and harvest index in the ratoon rice were significantly affected by year (Y), cultivation method treatment (T) and cultivar (C) (Table 6).The interactive effects of Y × T, Y × C, and Y × T × C were significant for total dry matter and harvest index in ratoon rice (Table 6). The interactive effect of T × C was not significant for biomass production or harvest index in ratoon rice. The total dry matter of ratoon rice in the CTDS and NTDS treatments was 8.5% and 9.6% higher, respectively, than in the CTTP treatment. The harvest index of ratoon rice in the CTDS and NTDS treatments were lower than in the CTTP treatment by 4.3% and 3.3%, respectively. The total dry matter and harvest index of ratoon rice in the NTTP and CTTP treatments were the same. On average, the rice hybrids had 18.7% higher total dry matter than the rice inbreds in the ratoon season; however, the harvest index was 1.2% lower for the rice hybrids than for the inbreds in the ratoon season. The total dry matter and harvest index of ratoon rice were the highest in 2017.

## Discussion

Our results show that there are significant differences in the main season yield, ratoon season yield, and annual yield among the four cultivation methods in the rice-ratoon rice cropping system (Table 3). Dong et al. found no differences in the main season yield, ratoon season yield, and annual yield between direct-seeded rice and transplanted rice in the rice-ratoon rice cropping system^14^. In contrast, Liu et al. reported that main season yield, ratoon season yield, and annual yield of direct-seeded and seedling broadcasting rice were significantly lower than the yields for manually transplanted seedlings^28^. In the present study, the main season yields in the CTDS and NTDS treatments were 2.8–6.1% higher than in the CTTP treatment, while the ratoon season yields in the CTDS and NTDS treatments were similar to or higher than the yields in the CTTP treatment. Consequently, CTDS and NTDS produced 3.2–4.4% higher annual yields than did CTTP. On average, a high annual yield of > 12 t ha^−1^ was achieved for CTDS and NTDS. The main season yields, ratoon season yields, and annual yields for rice grown in the NTTP and CTTP treatments were equal. These results suggest that CTTP can be replaced with CTDS and NTDS to maintain high rice grain yields and save on labor costs for rice production in the rice-ratoon rice cropping system.

The higher main season and ratoon season yields in the CTDS and NTDS treatments can be attributed to the higher sink size (spikelets per m^2^) and number of panicles per m^2^, which was significantly higher for CTDS and NTDS than for CTTP (Tables 4, 5). The high contribution of panicle number to grain yield in rice production has also been reported by Huang et al. and Lin and Luo^29,30^. However, these studies only analyzed the contribution of yield components to grain yield in single-season rice. Therefore, it is difficult to understand whether the contributions of yield components to grain yield vary with the rice-growing season. In the present study, the panicle number per m^2^ in the main rice crop and in ratoon rice for CTDS and NTDS was significantly higher than for CTTP, while the number of spikelets per panicle in the main rice crop and ratoon rice for CTDS and NTDS was lower than for CTTP. The average number of panicles per m^2^ in ratoon rice for the CTDS and NTDS treatments were 13.4–21.9% higher than in the CTTP treatment; which was associated with higher number of panicles per m^2^ in main rice crop for the CTDS and NTDS treatments compared with in the CTTP treatment. In other words, the adoption of direct-seeded rice can lead to higher number of panicles per m^2^ in main season and sufficient mother stems in ratoon season, resulting in high yields that resulted from higher in number of panicles per m^2^ in main crop-ratoon rice systems. The differences in grain filling and grain weight in the main rice crop and in ratoon rice among the four cultivation methods was relatively small or inconsistent. Therefore, improving panicle number per m^2^ is the key to increasing both main season and ratoon season yields in the rice-ratoon rice cropping system. The adoption of direct-seeded rice may also be a feasible approach towards achieving high number of panicles per m^2^ in the rice-ratoon rice cropping system.

In another approach, rice grain yield is determined by biomass production and harvest index^31^. However, it is generally thought that further improvements in rice grain yield might be driven by increasing the biomass production rather than the harvest index^32,33^, because there is little room to further increase the harvest index^34^. Consistently, in the present study, biomass production in the main rice crop and in ratoon rice in the CTDS and NTDS treatments was significantly higher than in the CTTP treatment; however, the harvest index of both the main and ratoon rice crops in the CTDS and NTDS treatments was significantly lower than in the CTTP treatment. This result indicates that further improvement in biomass production is important for improving rice grain yield while sustaining a high harvest index in the rice-ratoon rice cropping system. Biomass production can be increased by increasing dry weight per plant or plant number per unit land area, or both. In the present study, higher biomass production for both the main and ratoon rice crops in the CTDS and NTDS treatments compared to CTTP was mainly due to a higher plant number per unit land area, because the dry weight per plant of the main rice crop and ratoon rice in the CTDS and NTDS treatments was lower than in the CTTP treatment (data not shown). Our results indicate that direct-seeding is a feasible strategy to increase tiller numbers that results in high plant number per unit land area and high biomass production in the rice-ratoon rice cropping system.

The difference in main season, ratoon season, and annual rice yields between hybrid and inbred rice cultivars were significant in all four experiments. However, the magnitude of the differences in main season yield and ratoon season yield between the hybrid and inbred rice cultivars varied by crop establishment methods. The main season yield gap between hybrid and inbred rice cultivars was larger in transplanting treatment, but the ratoon season yield gap between the hybrid and inbred rice cultivars was larger in direct seeding treatment. The average main season and ratoon season yield of rice hybrids was significantly higher than that of rice inbreds, which was associated with longer growth duration, higher biomass and grain weight in rice hybrids compared with rice inbred (Table 3). The critical importance of biomass production to yield stability was previously emphasized by Jiang et al. and Zhang et al.^32,33^. The difference in main season yield between hybrid and inbred rice cultivars was mainly resulted from higher solar radiation during grain filling period (Table 2). However, the higher ratoon season yield in rice hybrids was associated with lower temperature during grain filling period compared with and rice inbred (Table 2). The number panicle per unit land area in ratoon rice mainly depended on the number of regenerated buds. Sprouting of axillary buds in ratoon rice requires certain conditions including temperature, light, humidity, and soil water content. Of all ecological conditions, the daily average temperature was the most important factor affecting regenerated buds growth; the duration of sunshine was second. The optimum temperature and relative humidity for sprouting axillary buds were 25–28 °C and the germination rate of axillary buds increased as the daily average temperature increased^35,36^. In the present study, the average panicles per m^2^ and spikelets per panicle in rice hybrids were lower than those in inbred lines in ratoon season. The difference in panicles per m^2^ and spikelets per panicle in ratoon season between hybrid and inbred rice cultivars were mainly resulted from the lower maximum and minimum temperatures and solar radiation in the ratoon crop growing season in rice hybrids, especially from harvest of the main rice crop to heading of the ratoon rice.

The average main season yield was 1.9–2.3 t ha^−1^ higher in 2017 than that in 2018–2020, which was associated with higher biomass and larger sink size that resulted from more panicles per m^2^ in 2017 compared with 2018–2020. The high maximum and minimum temperatures and solar radiation during tillering period (30 days after transplanting) of main rice crop might have contributed to the high panicles per m^2^ in 2017. More interesting, the sink size of main rice crop was larger in 2017 than in 2019 and 2020, though the grain filing was consistently higher in 2017 than 2019 and 2020 (Table 4). It is probable that lower solar radiation during grain filling period in 2019 and 2020 compared with 2017 reduced the grain filling of main rice crop^37^.

Rice hybrids not only had less variation in annual yield across four years, but also exhibited the smaller annual yield variability across management treatments compared with inbred lines. Annual yield in 2018, 2019 and 2020 was lower than in 2017 by 14.0–15.2% for rice hybrids and by 16.2–20.1% for inbred lines. Compared with CTTP, NTTP reduced annual yield by 2.0% and 1.0% for inbred lines and rice hybrids, respectively; CTDS and NTDS increased annual yield by 3.7–4.4% and 2.8–4.3% for inbred lines and rice hybrids, respectively. The simplified cultivation methods (NTTP, CTDS and NTDS) did not decrease or increase the annual yield of rice hybrids as much as in inbred lines. In other words, the rice hybrids was less sensitive to simplified cultivation methods than the inbred lines. Those results suggest that rice hybrids are more suitable than inbred cultivars to achieve high main season yields, ratoon season yields, and annual yields under simplified cultivation methods in southwest China. Similarly, previous studies have demonstrated that rice hybrids produce higher ratoon season yields and annual yields than do rice inbreds in direct seeded rice-ratoon rice cultivation systems^14,38^. The higher main season, ratoon season, and annual yields of rice hybrids compared to inbred cultivars can be attributed to the long growth duration and higher grain weight and biomass production, which suggests that developing cultivars with high grain weight and biomass production through breeding may also be a feasible approach to achieve high rice yield in the rice-ratoon rice cropping system in southwest China.

## Conclusion

Main season yields, ratoon season yields, and annual yields in the CTDS and NTDS treatments were higher than those in the CTTP treatment in the rice-ratoon rice cropping system in southwest China. The higher rice grain yields of direct-seeded rice in the CTDS and NTDS treatments were associated with higher panicle number per m^2^ and biomass production. This result indicates that CTTP can be replaced with CTDS and NTDS to maintain high rice grain yields and to save labor costs for rice production in the rice-ratoon rice cropping system in southwest China. Rice hybrids produced higher main season yields, ratoon season yields, and annual yields than inbred cultivars. The high grain yields produced by rice hybrids were attributed to long growth duration, high grain weight and biomass production. This suggests that breeding and selecting rice cultivars with high grain weight and biomass production may also be a feasible approach towards achieving high grain yields in the rice-ratoon rice cropping system in southwest China.

## Methods

A fixed field experiment was conducted in Fuji Town (29°10′ N, 105° 23′ E, 280 m asl) in Luxian County, Sichuan Province, China, from 2017 to 2020. The location has a subtropical zone humid climate with a mean annual temperature of 17.8 °C, mean annual rainfall of 1065 mm, mean annual relative humidity of 84%, mean annual sunshine of 1390 h, and an annual frost-free period of 341 days. The soil from the upper 20 cm surface layer contained 1.5 g kg^–1^ total nitrogen (N), 495.5 mg kg^–1^ total phosphorus, 31.6 g kg^–1^ total potassium, 30.3 g kg^–1^ organic matter, 150 mg kg^–1^ NaOH hydrolysable N, 4.4 mg kg^–1^ Olsen P, 162.0 mg kg^–1^ NH_4_OAc-extractable K, and had a pH of 4.3.

Four regionally popular and widely-adopted rice varieties, the hybrids ‘Nei6you103’ (NY103) and ‘Rongyou1015’ (RY1015) and the elite inbred cultivars ‘Huanghuazhan’ (HHZ) and ‘Jinnongsimiao’ (JNSM), were used in this experiment. Plant seeds were provided by the following organizations and persons: Rice and Sorghum Research Institute, Sichuan Academy of Agricultural Sciences and Rice Research Institute, Guangdong Academy of Agricultural Sciences. In each year, the hybrids NY103 and RY1015 and the elite inbreds HHZ and JNSM were grown using four cultivation methods: conventional tillage and transplanting (CTTP), conventional tillage and direct seeding (CTDS), no-tillage and transplanting (NTTP), and no-tillage and direct seeding (NTDS). Plots were arranged in a split-plot design with cultivation method as the main plot and rice cultivars as the sub-plots. Each treatment was replicated three times and the sub-plot size was 20 m^2^. The land preparation for the conventional tillage plots was carried out by plowing followed by a single harrowing, and for the no-tillage plots, herbicide was applied one week before planting. For transplanting, the seedlings were raised in a seedbed and 31- to 35-day-old seedlings were transplanted at a spacing of 26.4 cm × 20 cm with two seedlings per hill. For direct-seeding, pre-germinated seeds were broadcasted onto the soil surface at a seeding rate of 22.5 kg ha^−1^ on March 15th in each year. The fertilizers used were urea for nitrogen (N), single superphosphate for phosphorus (P), and potassium chloride for potassium (K) at doses of 225 kg N ha^−1^, 67.5 kg P_2_O_5_ ha^−1^, and 150 kg K_2_O ha^−1^. N was applied in four splits: 40% as basal, 20% at mid-tillering, 20% at panicle initiation, and 20% at 10 days full-heading of the main rice crop. P was applied as basal. K was applied in three splits: 50% as basal, 30% at panicle initiation, and 20% at 10 days full-heading of the main rice crop. Water management practices were as follows: water was drained off completely before sowing, maintaining soil saturation from sowing to the three-leaf stage of the main rice crop, after which the fields were kept flooded for the entire growing season. Insects, diseases, and weeds were intensively controlled throughout the entire growing season to avoid yield loss.

At the maturity stages of the main and ratoon crops, plants were sampled from an area of 0.48 m^2^ in the directed-seeded subplots and six hills in the transplanted subplots to determine the yield components, aboveground total biomass, and harvest index. After counting the number of panicles, the plants were separated into straw and grains by manual threshing. Filled spikelets were separated from the unfilled spikelets by submerging them in tap water. Both the filled and unfilled spikelets were then air dried. Three subsamples (30 g each) of the filled spikelets and all unfilled spikelets were counted to calculate the number of spikelets per panicle, the percentage of filled spikelets, and grain weight. After oven-drying to a constant weight at 70 °C, the dry weights of the straw and the filled and unfilled spikelets were determined. Aboveground total biomass was calculated as the sum of the total dry matter of the straw and the filled and unfilled spikelets. The harvest index was calculated as the ratio of filled grain dry weight to aboveground total biomass. Grain yield was determined from a 5-m^2^ area in the middle of each plot and adjusted to a moisture content of 14%.

The Statistix 8 software package (Analytical Software, Tallahassee, Florida, USA) was used for analysis of variance (ANOVA). The statistical model for ANOVA included replication, treatment (T), cultivar (C), year (Y), the two-factor interactions of T × C, T × Y, and C × Y, and the three-factor interaction of T × C × Y. The statistical significance was set at the 0.05 probability level.

### Ethical approval

The study complies with local and national regulations.

## Data Availability

All data generated or analysed during this study are included in the article.
